# Investigating the association between variability in sulcal pattern and academic achievement

**DOI:** 10.1038/s41598-022-15335-y

**Published:** 2022-07-19

**Authors:** M. Roell, E. Bellon, B. Polspoel, M. Declercq, B. De Smedt

**Affiliations:** Faculty of Psychology and Educational Sciences, Parenting and Special Education Research Group, Katholieke Universiteit Leuven, Leopold Vanderkelenstraat 32, 3000 Leuven, Belgium

**Keywords:** Neuroscience, Cognitive neuroscience

## Abstract

Investigating how the brain may constrain academic achievement is not only relevant to understanding brain structure but also to providing insight into the origins of individual differences in these academic abilities. In this pre-registered study, we investigated whether the variability of sulcal patterns, a qualitative feature of the brain determined in-utero and not affected by brain maturation and learning, accounted for individual differences in reading and mathematics. Participants were 97 typically developing 10-year-olds. We examined (a) the association between the sulcal pattern of the IntraParietal Sulcus (IPS) and mathematical ability; (b) the association between the sulcal pattern of the Occipito Temporal Sulcus (OTS) and reading ability; and (c) the overlap and specificity of sulcal morphology of IPS and OTS and their associations with mathematics and reading. Despite its large sample, the present study was unable to replicate a previously observed relationship between the IPS sulcal pattern and mathematical ability and a previously observed association between the left posterior OTS sulcal pattern and reading. We found a weak association between right IPS sulcal morphology and symbolic number abilities and a weak association between left posterior OTS and reading. However, both these associations were the opposite of previous reports. We found no evidence for a possible overlap or specificity in the effect of sulcal morphology on mathematics and reading. Possible explanations for this weak association between sulcal morphology and academic achievement and suggestions for future research are discussed.

## Introduction

Understanding how academic abilities, such as reading and math, develop but also mapping the various constraints, including biological factors, that may influence this development is of great importance for both science and society. This is especially so because research suggests that these academic abilities are important factors in determining career success, income and even psychological well-being^1^. Yet, the 2018 Program for International Student Assessment (PISA) results show that more than one in five pupils in the EU has insufficient proficiency in these key academic skills^2^. Investigating how the brain may constrain academic achievement is not only relevant to understanding brain structure but also to providing insight into the origins of individual differences in these academic abilities.

Research has extensively studied the function of key regions for processing mathematics and reading, namely bilateral IntraParietal Sulcus (IPS, see^3,4^ for a review) and the left Occipito-Temporal Sulcus (OTS) host of the Visual Word Form Area (VWFA)^5^ respectively. Much less studies have examined how individual differences in the structure of these regions are related to differences in academic performance^6–8^. Even less studies have investigated the long-term effect of early brain development on later mathematical and reading abilities. Specifically, such study of early cerebral constraints on math and reading would allow us to further unravel the question of causes of individual differences in academic learning. Recent studies have found evidence of associations between a marker of early brain development, sulcal morphology, and academic achievement, i.e. reading^9,10^ and mathematics^11^. The aim of this study was to replicate and extend these findings of an association between the sulcal morphology of the IPS and math abilities as well as the sulcal morphology of the OTS and reading.

Research has strived to delineate the brain regions functionally supporting mathematical ability. This body of literature converges to suggest that a fronto-parietal network is engaged during arithmetic in both children and adults^4,12,13^. Consistent across these data is the activation of the bilateral IPS during arithmetic^4,12^. Importantly, imaging studies in children have shown individual differences in arithmetic ability that are also reflected at the brain level. Specifically, children with low arithmetic fluency have been found to show a higher activity in, particularly the right IPS^14–16^. The activation of the IPS has also been consistently associated with basic number processing, such as symbolic number comparison^17–19^. IPS activation for symbolic numbers has been found to be cross-culturally consistent, as the IPS number-related activity has been found to be similar in Eastern and Western populations^20–23^. Importantly, studies have shown that over the course of development, symbolic number comparison abilities are associated with a progressive specialization of the IPS^18,24^. Children with developmental dyscalculia, a deficit in arithmetic and number processing^25^, have been found to have impairment in the IPS when processing number magnitudes and performing calculations. That is, the IPS in children with dyscalculia is not modulated in response to numerical processing demands to the same degree as in typically developing children^26,27^. It is important to note that in addition to the fronto-parietal network, the symbolic number processing network also comprises the right Inferior Temporal Gyrus (ITG), a region that has been dubbed the “number form area”^28–30^. The number form area is said to provide a crucial role in the symbolic number processing network as it is specialised in the visual processing of Arabic numerals^30^.

Studies have also investigated which brain structures are related to individual differences in mathematical ability, with the majority of studies focusing on the role of white and grey matter. Individual differences in math ability were found to be associated with higher fractional anisotropy, a parameter related to white matter microstructure, in white matter tracts connecting frontal lobes with basal ganglia and parietal regions^31–33^. Additionally, grey matter volume of the left IPS at the end of first grade has been found to be related to math competence a year later at the end of Grade 2^34^. Similarly, Evans et al.^6^ reported that grey matter volume of posterior parietal areas, including the left IPS, predicted the growth in arithmetic across primary school. However, in a recent study, Polspoel et al.^35^ examined grey matter volume using voxel-based morphometry, as did previous studies, as well as cortical complexity. Polspoel et al. did not find a significant association between children’s arithmetic fluency and the grey matter volume or the complexity of parietal regions such as the IPS.

Reading, on the other hand, systematically activates the left lateral OTS at a fixed location known as the Visual Word Form Area or VWFA^36^ relative to a reproducible mosaic of regions partially specialized for objects, faces, bodies and places^37^. The specialization of the VWFA site appears progressively as children start to learn to read^38^. Additionally, word-induced activation found at the site of the VWFA in good readers has been found to be cross-culturally consistent. Bolger et al.^39^ found that the peak activation of the VWFA in Japanese kana, Japanese logographic kanji, Chinese and roman alphabet readers was all within a millimeter of each other. Furthermore, children and adults with developmental dyslexia, a specific disorder of reading acquisition^25^, show an under-activation^40,41^ and a dysfunction in the VWFA whilst processing visual words^42^.

Studies have also examined the brain’s structure supporting reading, again focusing largely on white and grey matter data. In their study, Myers et al.^43^ found that increases in the volume of two left temporo-parietal white matter clusters were unique predictors of reading outcomes above and beyond family history, socioeconomic status and cognitive and preliteracy measure at baseline. Similarly, Niogi and McCandliss^44^ found a strong correlation between fractional anisotropy values in a left tempo-parietal white matter region and standardized reading scores of typically developing children. Additionally, they found that fractional anisotropy values in that region accounted for differences in reading score between typically developing children and children with dyslexia.

Turning to the role of grey matter, Altarelli et al.^45^ examined whether cortical thickness of the ventral occipitotemporal regions differed between children with dyslexia and typically developing children. They found a reduction in thickness in children with dyslexia compared with typically developing children in the VWFA, i.e., the Left posterior OTS. In their meta-analysis, Richlan et al.^46^ found converging evidence of reduced grey matter in the bilateral Superior Temporal Sulcus in participants with dyslexia compared to typically developing controls. They also found evidence of structural and functional abnormalities in the left occipitotemporal region in pre-readers with a family history of developmental dyslexia^46^.

One limitation of the existing body of data is that it does not furnish information on the influence of early cerebral constraints on academic achievement. Indeed, nearly all studies on the relation between neuroanatomy and academic achievement have focused on structural brain characteristics, such as grey matter density or white matter tracts^4,47^, that are affected by brain maturation and learning^48^. To evaluate early cerebral constraints on mathematics or reading achievement, it is important to examine neuroanatomical characteristics that are not affected by brain maturation and learning. Researchers have recently turned to the study of sulcal patterns of the brain as this *qualitative* feature of the cortex anatomy is determined in utero^49^ and is stable during development^50^. Studying brain sulcal patterns thus allows researchers to further unravel the question of causes of individual differences that are independent of learning and development.

Applying this methodology to the study of individual differences in mathematical cognition, a recent pre-registered study (https://osf.io/w3zvc) by Roell et al.^11^ examined whether the IPS sulcal pattern explained individual differences in number processing in sample of grade 1 to grade 4 children (*n* = 77; age 7.5–10.4 years) and adults (*n* = 21). They characterized the left and right IPS sulcal pattern as “sectioned” vs “not sectioned” based on the presence or absence of branches completely sectioning the IPS using Zlatkina & Petrides’^51^ classification. They found that IPS sulcal pattern explained part of the variance in both the children’s and adult’s symbolic number comparison and math fluency abilities but not in their non-symbolic number abilities. However, it is important to replicate the association between the sulcal morphology of the IPS and symbolic number processing and arithmetic ability in a larger sample of participants with a narrower age range.

Using the same method, Borst et al.^9^ and Cachia et al.^10^ have shown that the sulcal pattern of the OTS is associated with reading abilities. In their study, Borst et al.^9^ examined the relationship between OTS sulcal pattern and reading abilities in 8-year-old children (*n* = 16). They found that participants with an interrupted left OTS had significantly better reading abilities than participants with a continuous left OTS. Cachia et al.^10^, replicated this effect in a larger sample (*n* = 62) of adult participants and determined that this effect was specific to the posterior portion of the left OTS, which hosts the VWFA. They also found that the length of the OTS posterior interruption was positively correlated with reading skills. It remains to be determined whether the effect found by Cachia et al.^10^ of a specific association between the posterior portion of the left OTS and reading can also be replicated in a large sample of children.

In addition to studies that either focus on reading or on mathematics, it would be of interest to examine potential overlap or specificity of the sulcal pattern effect of the IPS on mathematical abilities and of the OTS on reading. Indeed, mathematical and reading abilities have been found to be correlated^52^. Comorbidity or co-occurrence of specific learning disorders in reading (dyslexia) and in math (dyscalculia) is remarkably high^53^. Furthermore, functional neural overlap of arithmetic and reading has been reported^54^. Children with dyslexia have been reported to show atypical brain activation patterns during arithmetic in arithmetic-related regions, such as the supramarginal gyrus^6^ and there is evidence to suggest that children with dyslexia and dyscalculia show highly overlapping patterns of brain activity during the processing of number^4^.

### Research aims

The aim of this study was threefold. For our first aim, Aim 1, we focused on the association between sulcal morphology of the IPS and individual differences in arithmetic and number processing**.** We wished to conceptually replicate our previous study^11^ in a larger dataset of participants in a more narrow age range. In continuation of Roell et al.’s study, we expected that IPS sulcal morphology explained part of the variability observed in arithmetic and symbolic number ability in typically developing children. That is, we hypothesized that children with a “sectioned” IPS would have greater symbolic number comparison and arithmetic abilities than children with a “not sectioned” IPS. Extending Roell et al.’s^11^ study, and to further our understanding of the relation between symbolic number processing, arithmetic and the sulcal morphology of the IPS, we investigated whether symbolic number processing mediated the association between sulcal morphology and arithmetic. Indeed, relations between children’s mathematics achievement and their basic number processing skills have been reported in both cross-sectional and longitudinal studies^18,55,56^. In their longitudinal study Bartelet et al.^57^ reported that symbolic number processing was consistently a significant predictor of arithmetic achievement regardless of children’s level of arithmetic proficiency. Similarly, a significant correlation between the activation in the IPS during symbolic number task and arithmetic task has been found^58^. Moreover, Roell et al.^11^ observed an association of sulcal morphology with both number processing and arithmetic ability. As such, it would appear interesting to examine whether the relation between IPS sulcal morphology and arithmetic ability is mediated by symbolic number processing. We hypothesized that symbolic number processing mediated the association between IPS sulcal morphology and arithmetic ability. That is, participants with a “sectioned” IPS would have greater symbolic number comparison abilities; these greater symbolic number comparison abilities would in turn be associated with greater arithmetic abilities.

Our second aim, Aim 2, was to conceptually replicate and extend the findings of Cachia et al.^10^ and Borst et al.^9^ in a larger sample of children. Specifically, we examined whether the effect found by Cachia et al.^10^ in adults of a specific association between the posterior portion of the left OTS and reading can also be replicated in children. Namely, we expected that participants with an interrupted Left OTS, in particular in its posterior portion hosting the VWFA, have better reading performance than participants who have a continuous Left OTS.

Our third aim, Aim 3, was to examine whether the effect of sulcal morphology on academic abilities is specific. As discussed above, studies examining behaviour, learning disorders and functional networks point towards an overlap between reading and mathematical abilities. Additionally, the specificity of the IPS for number and arithmetic processing^17^ and the VWFA for reading^59^ remains controversial. As such, in view of a potential overlap, it becomes relevant to determine whether the effect of the IPS sulcal pattern on numerical abilities is specific, as well as to verify whether the effect of the OTS sulcal pattern on reading is specific. The overlap and specificity of the IPS and OTS was examined through three different analyses. Firstly, we tested whether the sulcal pattern of IPS predicted reading and the sulcal pattern of left posterior OTS predicted arithmetic abilities. Secondly, we ran the same analyses as in Aim 1 and 2 but we added the other academic ability as a covariate. That is, we examined the effect of IPS sulcal morphology on mathematical ability whilst controlling for reading ability. Similarly, we examined the effect of OTS sulcal morphology on reading ability whilst controlling for mathematical ability. Thirdly, we used a third sulcal pattern, which is much less related to reading or mathematical ability, as a control region. More specifically we examined if the sulcal morphology of the Anterior Cingulate Cortex (ACC) was related to math and reading abilities. Studies have found that an asymmetrical ACC sulcal pattern, that is each hemisphere had a different ACC sulcal pattern, was associated with higher inhibitory control efficiency in both children^9,10^ and adults^50,60,61^. Against this background, we predicted that, if the association between sulcal patterns and academic achievement is specific, this sulcal pattern of the ACC will be much less related to math abilities or to reading ability. Finally, we also tested whether a relationship between the sulcal pattern of the IPS and that of the OTS could be found. That is, are participants with an “interrupted” OTS more likely to have a “sectioned” IPS and vice-versa?

To address the three aims outlined above, we utilized structural MRI data from two existing datasets collected in the same age range. These structural data were previously collected for a variety of different studies^62,63^ and all had the same structural image scans as well as academic achievement measures (Tempo Test Arithmetic task, symbolic number comparison task, Dutch one-minute reading test). We selected behavioural measures common to all datasets that measured academic achievement. It is of note that both datasets initially focused on mathematical ability. As such, the datasets contained more mathematical tasks which allowed us to also investigate the cognitive correlates of mathematical ability (through our mediation analysis) in a more fine-grained way as compared to reading ability. The functional data has already been published^62,63^ and 47 of the structural MRI (grey matter volume and DTI) data has already been published^35,64^. However, no research has been conducted on the sulcal morphology data so far.

Because sex has been shown to have a potential effect on sulcal anatomy^65^ and because intellectual ability (IQ) has been found to be related mathematical abilities^66^ and reading abilities^67^, we accounted for potential effects of both sex and IQ on the relation between sulcal morphology and academic skills. Similarly, given that we combine data from two different datasets, we controlled for the potential effect of datasets.

## Methods

### Participants

Our combined dataset included participants from two separate datasets comprising a total *N* of 97 typically developing grade 4 children with a wide range of mathematical abilities. The pre-registration protocol initially expected to use a third dataset. However, the T1 MRI data had too much movement for the BrainVisa software to read it, and the dataset was therefore excluded from the study (see Supplementary Sect. S3). Data from both datasets were all collected with the same scanner in a similar time window at KU Leuven University in Leuven, Belgium. All participants were Grade 4 children collected from communities within and around Leuven. From these datasets 47 participants were drawn out from Polspoel et al.^64^ and 50 from Bellon et al.^62^. T1 MRI data that had too much movement for a clear labeling of the IPS and OTS were excluded, as were T1 MRI data for which the two raters were not able to come to a final classification agreement (see below for more information). For the IPS analyses, we excluded 4 participants (2 due to classification disagreements) from Polspoel et al.^64^ and 3 (1 due to classification disagreements) from Bellon et al.^62^. This resulted  in the dataset of 90 (47 female) with a mean age of 9.94 ± 0.39 years. For the OTS analyses, we excluded 14 participants (6 due to classification disagreements) from Polpsoel et al.^64^ and 17 participants (8 due to classification disagreements) from Bellon et al.^62^. This resulted in a dataset of 66 participants (30 female) with a mean age of 9.94 ± 0.38 years. Informed consent was obtained from all participant’s caregivers. All participants were tested in accordance with national and international norms governing the use of human research participants.

### Behavioral measures

Arithmetic fluency was measured with the Tempo Test Arithmetic (TTA)^68^, a standardized test of arithmetic fluency similar to the Math Fluency subtest of the Woodcock-Johnson III^69^. The test consisted of five columns of arithmetic items (one column per operation and a column with mixed operations) which increased in difficulty. Each participant was given a minute per column/subtest to provide as many correct answers as possible. The score on this test combined the number of correctly solved problems on each subtest within the time limit. This test combined speed and accuracy into one index score.

A computerized assessment of number processing ability was also used, a symbolic number comparison task^70^. In this task, children had to compare two simultaneously presented Arabic digits, displayed on either side of a 15-inch computer screen. They had to indicate the larger one by pressing a key on the side of the larger digit. Stimuli comprised a combination of numbers 1–9 thus yielding a total of 72 trials. The position of the largest number was counterbalanced. Each trial started with a central fixation point of 200 ms followed by a blank screen of 800 ms. Thereafter stimuli appeared and remained visible until response. Response times and answers were registered. To familiarize children with the task, two practice trials were presented beforehand. We chose to focus on the reaction time of the participants during the single-digit symbolic number comparison task, as the accuracy of the Grade 4 participants was high, with a mean of 97.16 ± 2.40% (see Table 1), thereby resulting in a ceiling effect. Additionally, in their meta-analysis Schneider et al.^56^ observed that with older participants, reaction time is the most reliable measure.

**Table 1:** Descriptive statistics of the academic measures and IQ per dataset.

		Arithmetic ability	Symbolic number ability (reaction time)	Symbolic number ability (accuracy)	Reading ability	IQ (WISC-III or Raven)
Polspoel et al.^64^	M	100.6	778.3	98.1	61.8	44.2
	SD	16.9	123.5	1.9	10.5	10.7
	Minimum	73.0	567.7	93.1	39.0	24.0
	Maximum	136.0	1058.89	100.0	90.0	69.0
Bellon et al.^62^	M	99.6	761.6	96.2	60.0	36.4
	SD	19.1	127.3	2.5	11.9	7.8
	Minimum	65.0	561.9	91.7	37.0	14.0
	Maximum	142.0	1088.42	100.0	87	49.0
Both datasets	M	100.1	769.5	97.2	60.9	40.1
	SD	17.9	125.0	2.4	11.2	10.0
	Minimum	65.0	562.0	91.7	37.0	14.0
	Maximum	142.0	1077.4	100	90.0	69.0

Reading ability was measured by the normed and standardized Dutch One-Minute Test^71^ which is similar to the Woodcock Johnson reading fluency measure and is widely used in Flanders. For this test participants are told to correctly read aloud for 1 min as many words as possible in the list of 116 words. The list of words consists of one up to five syllable words of increasing difficulty. Similarly to the TTA, the test combines speed and accuracy into one index score.

Intellectual ability (IQ) was assessed by either the WISC-III-NL block design^72^ for the Polspoel et al.^64^ or the Raven Progressive Matrices^73^ for the Bellon et al.^62^. In both cases, raw scores were used.

### MRI acquisition

Structural (T1) MRIs were acquired with a 3 T Philips Ingenia CX Scanner at the Department of Radiology at the University Hospital of Leuven, Belgium. High-resolution T1-weighted anatomical images (182 slices, resolution 0.98 × 0.98 × 1.2 mm, TR = 9.6 ms TE = 4.6 ms, 256 × 256 acquisition matrix) that were obtained for all participants. These MRIs were adapted for sulcus segmentation required for the three-dimensional reconstruction of the fine individual cortical folds.

### Sulcal morphology

Analysis of the OTS and IPS sulcal morphology was performed using BrainVISA 4.5 software (http://brainvisa.info/). The sulcal patterns of the IPS and OTS were visually assessed using three-dimensional, mesh-based reconstruction of cortical folds. All MRI data were anonymized, and manual labeling of IPS and OTS in left and right hemispheres was carried out blind to possible confounding information, i.e. participant’s age, reading, arithmetic and symbolic number comparison abilities, as well as the label of the sulcal pattern in the contralateral hemisphere. Importantly, the manual labeling of the IPS and OTS was carried out independently by two experimenters with 86.3% agreement and Cohen’s kappa of 0.77 for the right hemisphere and 75.5% agreement and Cohen’s kappa of 0.65 for the left hemisphere.

#### IPS

**Figure 1:**
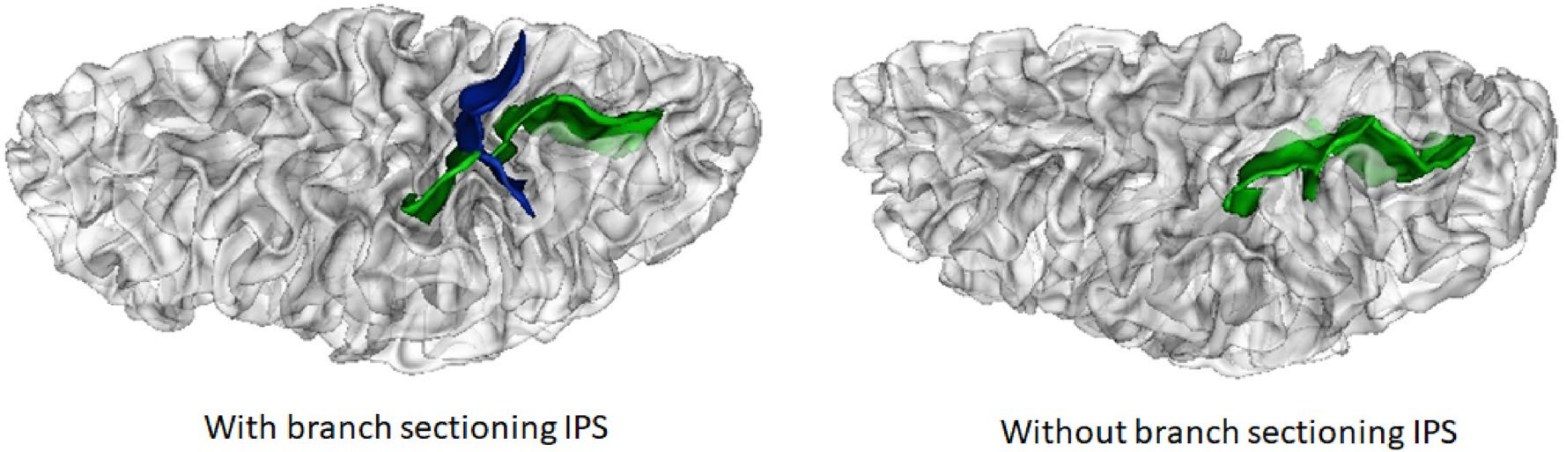
Sulcal patterns of the IPS. Example of a “Sectioned” (on the left) IPS and a “Not Sectioned” IPS (on the right). The IPS sulcus is depicted in green and the branch completely sectioning the IPS is depicted in blue.

We used the protocol put forward by Roell et al.^11^ to classify the IPS. Following the atlas by Zlatkina and Petrides^51^, we used the Central Sulcus and the Postcentral Sulcus as landmarks to localize the horizontal segment of the IPS extending posterior to these sulci and as far as the sulcus of Brissaud and the anterior part of the para-occipital sulcus^51^. This anatomical identification was cross-validated using functional information based on the mean positions of the activation coordinates for symbolic and non-symbolic number processing: X = − 34  Y = − 48 Z = 44) for left IPS and X = 36 Y = − 46 Z = 44) for right IPS. These coordinates were derived from a meta‐analysis of 57 studies^74^. Right and left IPS sulcal pattern were then classified as “Sectioned” or “Not Sectioned” based on the presence or absence of branches completely sectioning the IPS (see Fig. 1).

#### OTS

**Figure 2:**
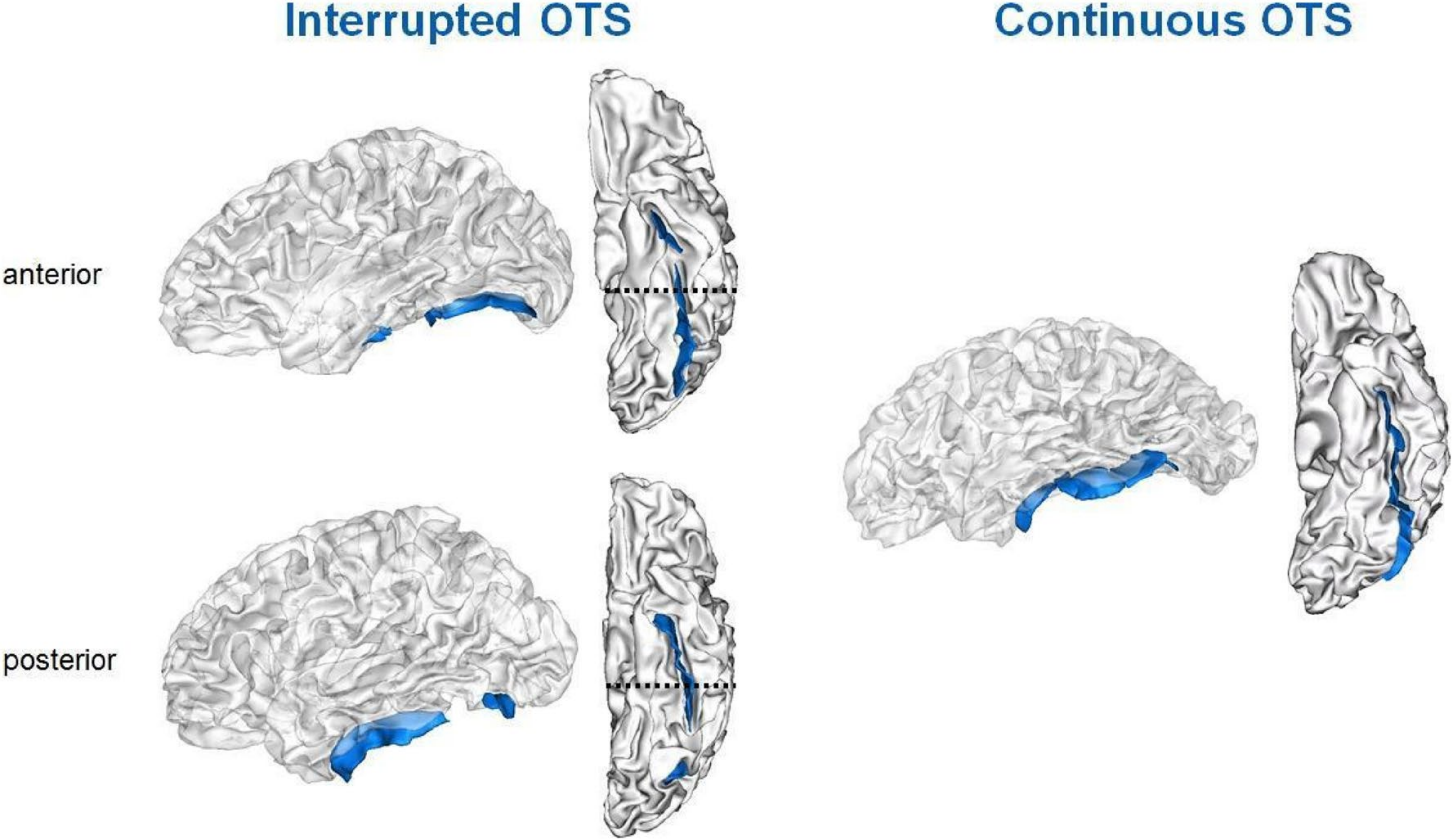
Examples of different OTS (depicted in blue) sulcal patterns, the “interrupted” OTS on the left and the “continuous” on the right. Interrupted OTS were further classified as anterior (top left) and posterior (bottom left). The posterior extremity of the brainstem (dashed line) was used as a limit to define the anterior and posterior interruptions of the OTS.

For the characterization of the OTS, we used exactly the same protocol put forward by Cachia and colleagues^10^. The sulcal pattern of the left and right OTS was characterized as “interrupted” when the OTS had interruptions and “continuous” otherwise. In addition, to investigate the possible effect of the position of the OTS interruption, we identified whether the OTS interruption was located in the posterior part of the sulcus, host of the VWFA in the left hemisphere^5^, or in the anterior part of the sulcus (see Fig. 2). We used an anatomical criterion, namely the Y-coordinate of the posterior extremity of the brainstem as a limit to define the anterior and posterior interruptions of the OTS. The functional validity of this anatomical criterion has already been established^10^.

#### ACC

**Figure 3:**
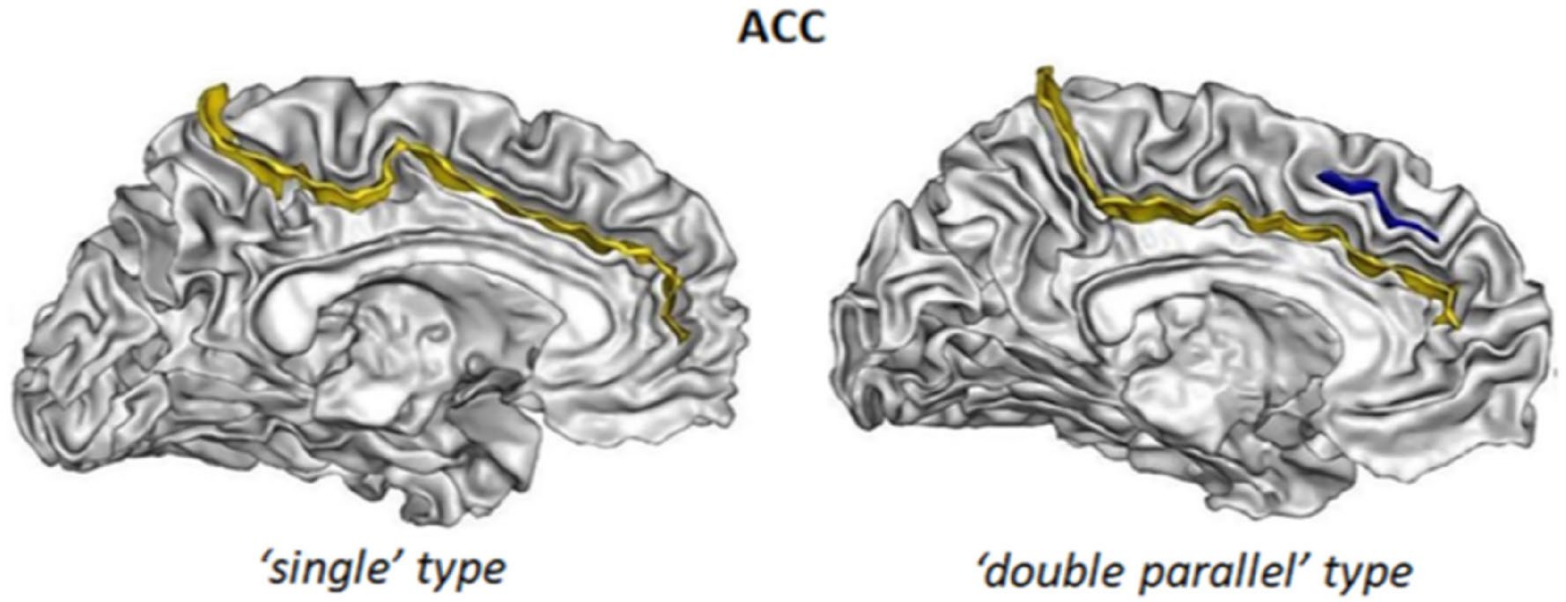
Example of different ACC sulcal patterns. The “single type”, with only the cingulate sulcus (depicted in yellow) and the “double parallel type”, with an additional PCS (depicted in blue).

We followed the protocol elaborated by Cachia and colleagues^50,75–77^ to classify the ACC. The ACC sulcal pattern was categorized in two types: “single type” or “double parallel type”^78^ depending on the absence or presence of a paracingulate sulcus (PCS), which is a variable secondary sulcus^79^, see Fig. 3. The PCS was defined as located dorsal to the cingulate sulcus with a course clearly parallel to the cingulate sulcus^79,80^. To reduce ambiguity from the confluence of the PCS and the cingulate sulcus with the superior rostral sulcus, we determined the anterior limit of the PCS as the point at which the sulcus extended posteriorly from an imaginary vertical line running perpendicular to the line passing through the anterior and posterior commissures (AC-PC) 78. The PCS was considered absent if there was no clearly developed horizontal sulcal element parallel to the cingulate sulcus and extending at least 20 mm (interruptions or gaps in the PCS course were not taken into account for the length measure). PCS length was measured in a standard (MNI) space.

#### Analysis

A statistical analysis plan for this study was pre-registered on the Open Science Framework (https://osf.io/cqsu9). We have modified part of our statistical approach upon review (see Supplementary Sect. S3). Analyses were performed using the R^81^ and JASP software^82^. For each of the analyses, we reported effect size in terms of Cohen’s d for *t*-tests and eta-square for ANOVAs. Bayesian analysis were also used to quantify the evidence against (BF_10_) or for (BF_01_) the null hypothesis.

Before conducting analyses relevant to the 3 aims, we first assessed whether dataset, sex and IQ were associated with the academic measures.

### Aim 1: associations between arithmetic/symbolic number and IPS sulcal morphology

For our first aim, we determined whether the sulcal patterns of the right and left IPS were associated with the participant’s arithmetic and symbolic number processing abilities.

We first assessed whether the sulcal patterns of the right and left IPS were associated with arithmetic and symbolic number processing ability. We performed analyses of covariance (ANCOVA) based on a linear model including right and left IPS sulcal pattern with sex, dataset and IQ as covariates.

**Figure 4:**
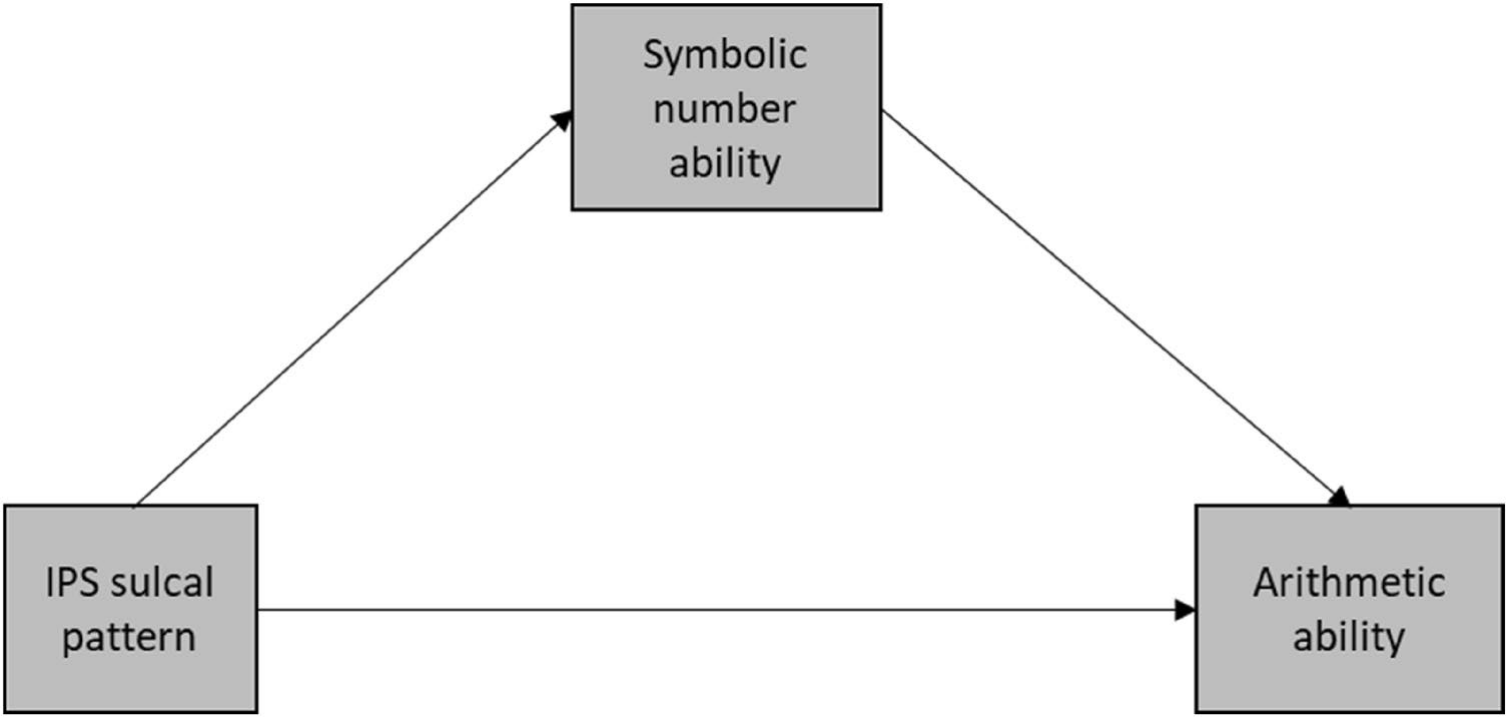
Mediation model for the relationship between IPS sulcal pattern and arithmetic ability as mediated by symbolic number ability.

Next, we assessed whether participants’ arithmetic scores were correlated with the symbolic number comparison. If these were indeed correlated and we found an association between IPS and arithmetic abilities, we ran a mediation model, to examine whether symbolic number processing mediated the relation between IPS sulcal morphology and arithmetic ability. We hypothesized a direct effect between IPS sulcal pattern and arithmetic ability as well as an indirect effect between IPS sulcal pattern and arithmetic ability through symbolic number abilities, see Fig. 4.

### Aim 2: associations between reading and sulcal morphology OTS

For our second aim, we strived to replicate the results found by Borst et al.^9^ and Cachia et al.^10^. To do so, we first assessed the relationship between OTS sulcal pattern (left posterior OTS, left anterior OTS, right posterior OTS and right anterior OTS) and reading. We performed analyses of covariance (ANCOVA) based on a linear model including OTS sulcal pattern (left posterior OTS, left anterior OTS, right posterior OTS and right anterior OTS) with as sex, dataset and IQ as covariates.

If we found that participants with interrupted left posterior OTS had better reading skills than participants with a continuous left posterior OTS, we then would examine whether we may find a positive correlation between the length of the interruption and reading ability, as was found in^10^.

### Aim 3: overlap and specificity of sulcal morphology related to mathematics and reading

Three analyses were performed to evaluate the specificity of the effect of the IPS sulcal pattern on mathematical abilities and of the effect of the OTS sulcal pattern on reading abilities. Firstly, we investigated whether the sulcal pattern of the IPS might be related to reading ability and whether the sulcal pattern of the OTS might be related to mathematical ability via *t*-tests. Secondly, we tested whether the association between IPS sulcal pattern and mathematical ability remained when reading ability was controlled for. Likewise, we examined whether the association between OTS sulcal pattern and reading ability remains when mathematical ability was  controlled for. Thirdly, we examined whether mathematical and reading ability were associated with the sulcal pattern of the ACC, a sulcus unrelated to both academic skills. To do so, we investigated whether ACC pattern groups (“single type” vs “double parallel type”) differed in their mathematical and reading ability. Finally, we also tested whether a relation between the sulcal pattern of the IPS and that of the OTS could be found. That is, whether participants with an “interrupted” OTS were more likely to have a “sectioned” IPS and vice-versa.

### Ethical approval

The study was approved by the Social and Societal Ethics Committee of the KU Leuven. All methods were carried out in accordance with relevant guidelines and regulations.

### Informed consent

Written informed consent was obtained from each subject prior to testing.

### Consent for publication

Participants provided written informed consent for the publication of any associated data.

## Results

### Preliminary analyses

We first explored the data for potential outliers, defined as values larger or smaller than 1.5 times the interquartile range. Four outliers were identified and excluded in the symbolic number measure. In the arithmetic measure, one outlier was identified and excluded. In the reading measure, two outliers were identified and excluded. Notably, our data was analyzed both with and without the outliers. The inclusion of the outliers did not affect the results.

We then analyzed whether sex and IQ were associated with arithmetic, symbolic number and reading abilities. Sex was not associated with arithmetic abilities, *t*(87) = − 1.65, *p* = .10, Bayesian analysis weakly supported the null hypothesis^83^, BF_01_ = 1.37. Sex was also not associated with symbolic number abilities, *t*(83.62) = 0.73, *p* = *.4*6, and the evidence for this absence of an association was weak BF_01_ = 1.94. Similarly, sex was not associated with reading, *t* (62) = 0.01, *p* = .98, and the evidence for this absence of an association was substantial BF_01_ = 3.91. IQ was not correlated with arithmetic ability, *r*(87) = 0.10, *p* = .30, symbolic number abilities *r*(84) = − 0.05, *p* =  .64, or reading, *r*(62) = 0.03, *p* =  .78. Additionally, Bayesian analysis substantially supported the null hypothesis^81^ for the absence of an association between IQ and arithmetic, BF_01_ = 4.53, IQ and symbolic number abilities, BF_01_ = 6.67, and for IQ and reading, BF_01_ = 6.18. To conclude, neither sex nor IQ were associated with the academic skills measured in this study.

We also analyzed whether there were any dataset differences in arithmetic, symbolic number comparison and reading abilities (see Table 1). No dataset differences were found in arithmetic abilities, *t*(87) = 0.26, *p* = .79, and the evidence for the absence of a difference was substantial BF_01_ = 4.37. No dataset differences were found in symbolic number comparison abilities, *t*(84) = 0.61, *p* = .53, and the evidence for the absence of a difference was substantial BF_01_ = 3.76. No dataset differences were found in reading abilities, *t*(62) = 0.62, *p* = .52, and the evidence for the absence of a difference was substantial BF_01_ = 3.29.

We compared the distribution of the sulcal patterns in the current study (Table 2) with the distributions reported in earlier studies^10,11^. We did not observe a difference in the frequency distribution in the sulcal pattern of the left IPS in the current study and the sulcal pattern of the IPS of Grade 2–4 participants in Roell et al.^11^ for the left IPS *χ* = 2.45; *p* = .11. The distribution of the right IPS was found to differ slightly, *χ* = 4.36; *p* = .04. No frequency distribution differences between the sulcal pattern of the OTS in the current study and the sulcal pattern of the OTS in Cachia et al.^10^, were found for the left posterior OTS *χ* = 0.56; *p* = .48, the right posterior OTS *χ* = 0.16; *p* =.75, and right anterior OTS *χ* = 2.01; *p* = .16. However, the distribution of the left anterior OTS differed between the current study and Cachia et al.^10^, *χ* = 25.36; *p* < 0.0001. No frequency distribution difference between the sulcal pattern of the ACC in the current study and the sulcal pattern of the ACC in Tissier et al.^50^, was found for the left ACC *χ* = 3.70; *p* = .06 and the right ACC *χ* = 0.05; *p* = .83.

**Table 2:** Frequency distribution of the sulcal pattern of the IPS, OTS and ACC in the current study and in previous studies.

			Current study	Previous study	Current vs. Previous study
IPS (Grade 2–4; Roell et al.^11^)	Left	Sectioned	32 (36%)	26 (34%)	*χ* = 2.45; *p* = .11
		Not sectioned	58 (64%)	51 (66%)	
	Right	Sectioned	40 (44%)	32 (63%)	*χ* = 4.36; *p* = .04
		Not sectioned	50 (56%)	19 (37%)	
OTS (Cachia et al.^10^^)^	Left Posterior	Interrupted	21 (32%)	20 (39%)	*χ* = 0.56; *p* = .48
		Continuous	45 (68%)	32 (62%)	
	Left Anterior	Interrupted	10 (15%)	31 (60%)	*χ* = 25.36; *p* < .0001
		Continuous	56 (85%)	21 (40%)	
	Right Posterior	Interrupted	21 (32%)	18 (35%)	*χ* = 0.16; *p* = .75
		Continuous	45 (68%)	34 (65%)	
	Right Anterior	Interrupted	22 (33%)	24 (46%)	*χ* = 2.01; *p* = .16
		Continuous	44 (67%)	28 (54%)	
ACC (Tissier et al.^50^)	Left	Single	32 (36%)	7 (18%)	*χ* = 3.70; *p* = .06
		Double	58 (64%)	31 (82%)	
	Right	Single	54 (60%)	22 (58%)	*χ* = 0.05 ; *p* = .83
		Double	36 (40%)	16 (42%)	

### Aim 1: associations between arithmetic/symbolic number and IPS sulcal morphology

**Figure 5:**
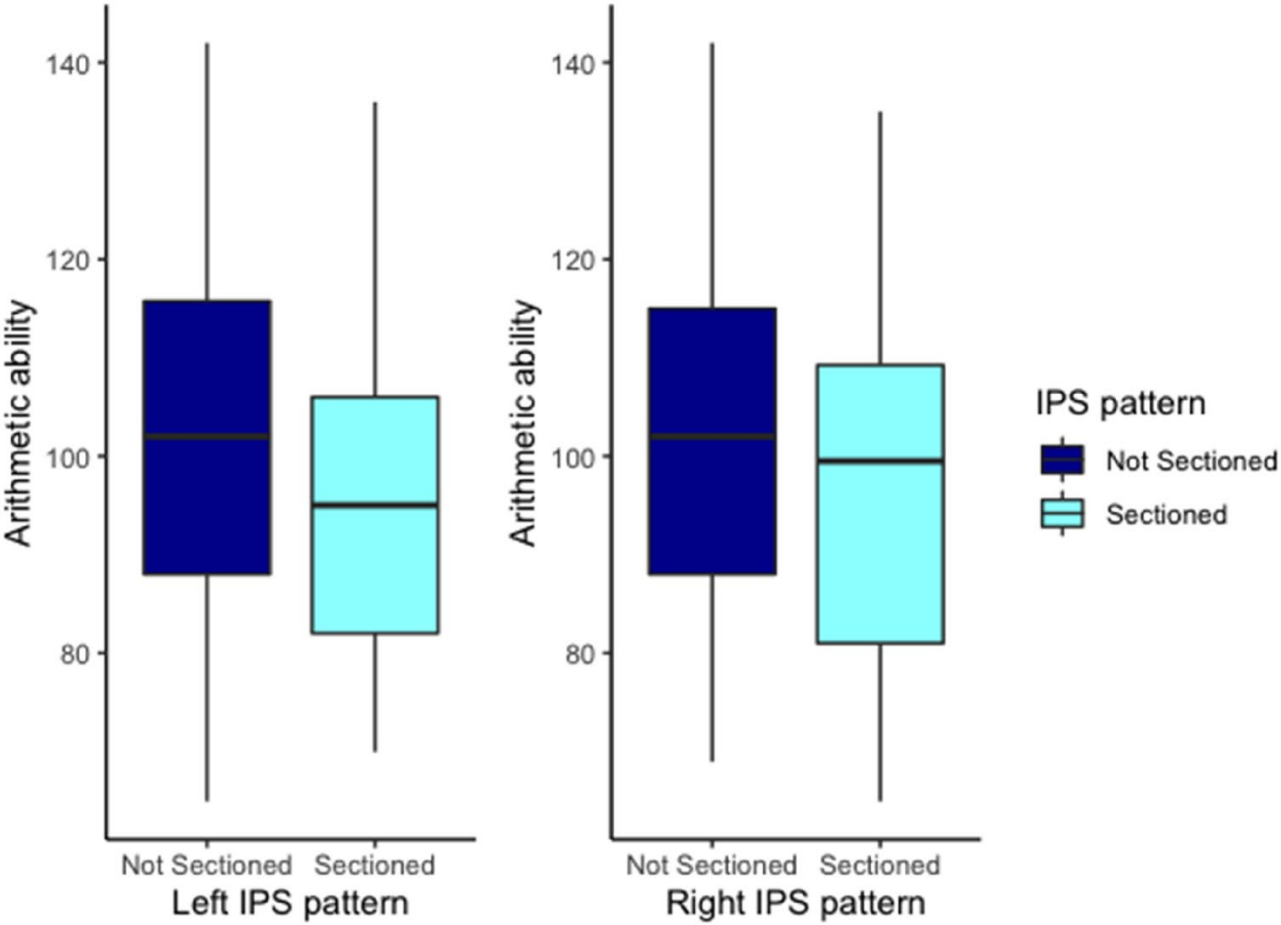
Effect of the left and right IPS pattern on arithmetic ability.

We first examined whether the sulcal pattern of the left and right IPS was associated with arithmetic abilities controlling for dataset, IQ and sex. An association between left IPS and arithmetic, *F*(1,77) = 4.29, *p* = .04, was found but the evidence for this association was weak BF_10_ = 2.64, see Fig. 5. Contrastingly, no association between right IPS and arithmetic was found, *F*(1,77) = 1.65, *p* = 0.20, and the evidence for the absence of an association was weak BF_01_ = 1.55, see Fig. 5. The results on the covariates IQ, Sex and dataset are provided in Supplementary Sect. S4.

**Figure 6:**
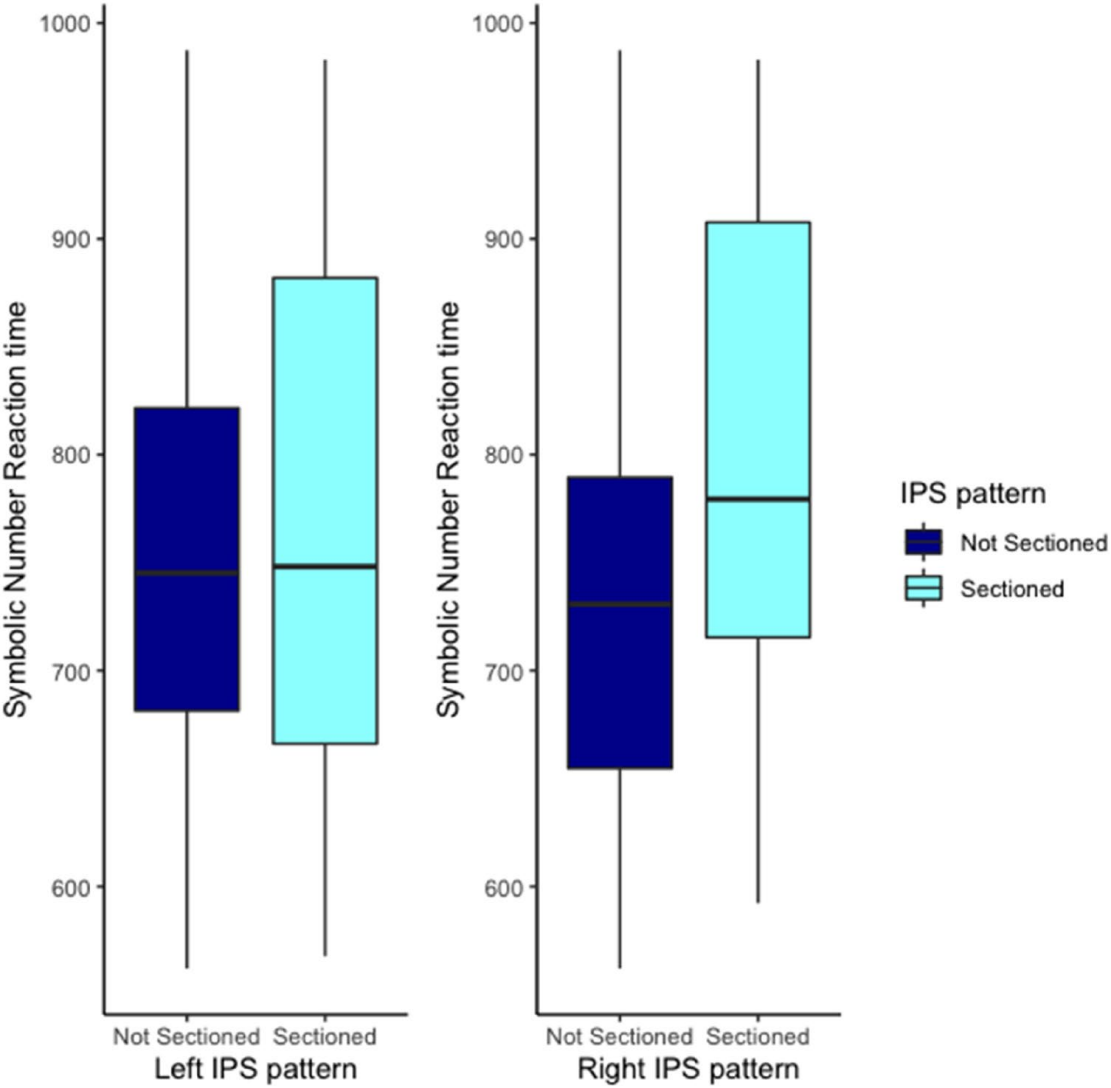
Effect of the left and right IPS pattern on symbolic number reaction time (in ms).

We then analyzed whether the sulcal pattern of the left and right IPS was associated with symbolic number ability controlling for dataset, IQ and sex. The left IPS was not found to be significantly associated with symbolic number abilities, *F* < 1, and the evidence for this absence of an association was substantial BF_01_ = 3.67. Right IPS was significantly associated with symbolic number ability, *F*(1,74) = 4.70, *p* = *.0*3, and the evidence for this association was substantial BF_10_ = 4.13. Participants with a sectioned Right IPS had significantly longer reaction times in the symbolic number comparison task than participants with a not sectioned IPS, see Fig. 6. The results on the covariates IQ, sex and dataset are provided in Supplementary Sect. S4.

Next, we assessed whether participants’ scores in the arithmetic task were correlated with the symbolic number comparison task. Arithmetic ability was found to be correlated with symbolic number ability, *r*(83) = − 0.35, *p* < 0.001 and the evidence for this association was very strong with BF_10_ = 44.70. Although arithmetic ability and symbolic number ability were indeed correlated, we did not find an association between IPS and mathematical abilities (arithmetic and symbolic ability). As such, it was not appropriate to conduct mediation analyses examining whether symbolic number processing mediated the relation between IPS sulcal morphology and arithmetic ability (but see Supplementary Sect. S1).

### Aim 2: associations between reading and sulcal morphology OTS

**Figure 7:**
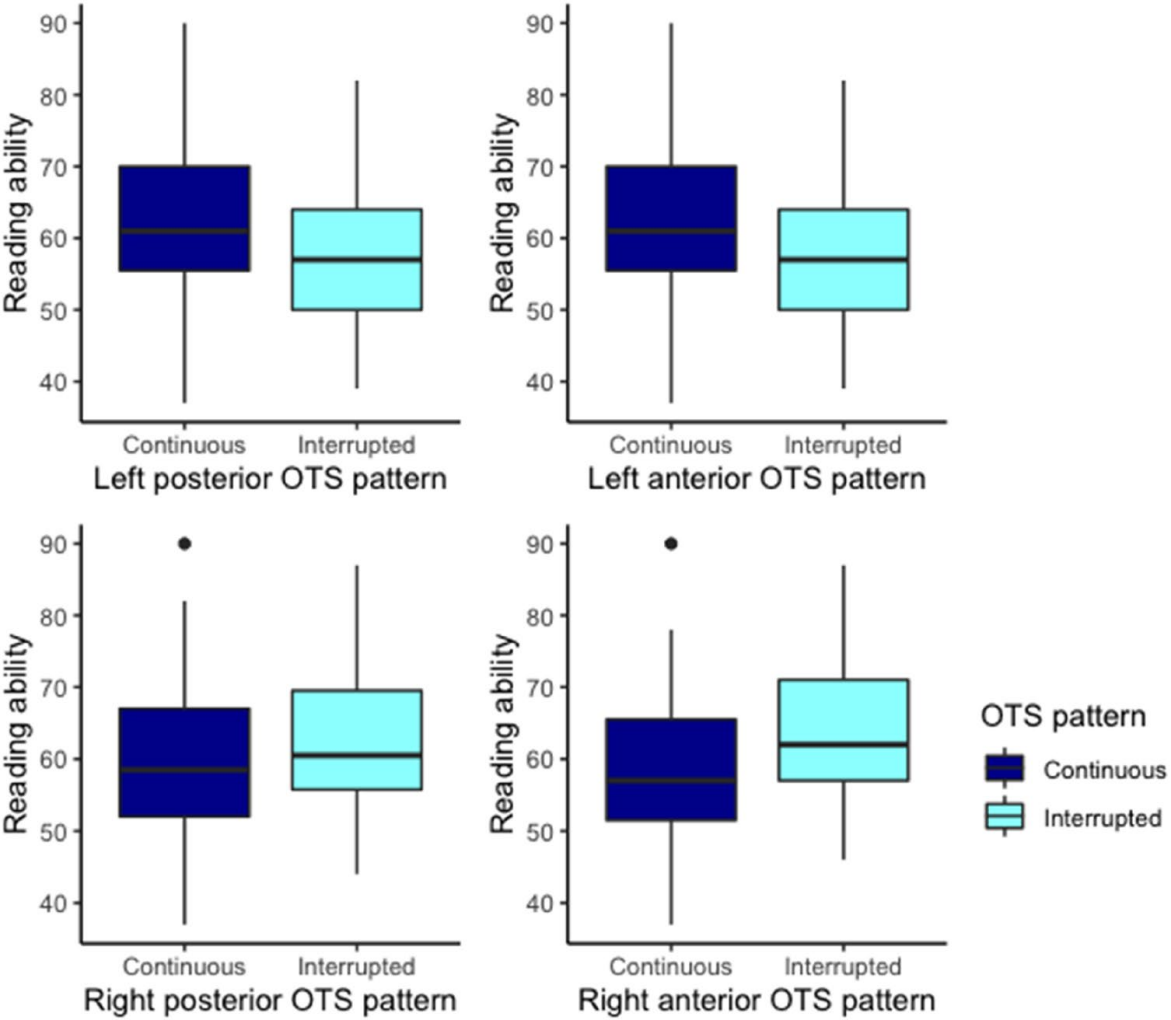
Effect of the four OTS patterns (left posterior OTS, left anterior OTS, right posterior OTS and right anterior OTS) on reading ability.

The left posterior OTS was significantly associated with reading ability *F*(1,44) = 6.15, *p* = 0.01 and evidence for this association was substantial, BF_10_ = 5.39. Left anterior OTS was not significantly associated with reading ability, *F*(1,44) = 1.16, *p* = .28, and evidence for the absence of this association was substantial BF_01_ = 3.43. The right posterior and the right anterior OTS were not significantly associated with reading ability, respectively *F*(1,44) = 2.77, *p* = .10 and *F*(1,44) = 2.99, *p* = .09 (see Fig. 7). The evidence for this absence of an association was weak for right anterior OTS BF_01_ = 1.59 and substantial for right posterior OTS BF_01_ = 4.33. The results on the covariates IQ, sex and dataset are provided in Supplementary Sect. S4.

### Aim 3: overlap and specificity of sulcal morphology related to mathematics and reading

Our third aim was to examine the specificity of the effect of the IPS sulcal pattern on numerical abilities as well as the specificity of the effect of the OTS sulcal pattern on reading. However, this study failed to find a clear effect of the IPS sulcal pattern on arithmetic and symbolic number comparison abilities, nor did the study find a clear effect of the OTS sulcal pattern on reading abilities. As such, it seemed ill-guided to study the specificity of both these effects (but see Supplementary Sect. S2).

## Discussion

The present study sought to investigate the impact of neuroanatomical constraints on key academic abilities, namely mathematical and reading ability. We examined a specific neuroanatomical characteristic that is not affected by brain maturation and learning, namely the sulcal patterns of the brain^49,77^. Specifically, we focused on the sulcal pattern of the IPS and the OTS as both regions have consistently been associated with mathematical processes and reading respectively^3–5^. Through this approach we wished to gain some insight as to biological constraints that influence the development of reading and mathematics.

The first aim focused on the relationship between the IPS sulcal pattern and mathematical ability. We hypothesized in accordance with Roell et al.’s^11^ findings, that IPS sulcal morphology would explain part of the variability observed in symbolic number comparison and arithmetic tasks in typically developing children. In contrast to our expectations, no significant association between arithmetic ability and IPS sulcal morphology (left and right) were found. A significant association was found between the right IPS sulcal morphology and symbolic number abilities. However, this association was weak. Furthermore, this association was the opposite to what we had hypothesized and what Roell et al.^11^ found. We found that participants with a “sectioned” right IPS had worse symbolic number abilities, here reaction time, than participants with a “not sectioned” right IPS. Importantly, we were able to replicate the well-known association between symbolic number ability and arithmetic ability, hereby assuring the validity of both measures.

The second aim of this study centered on the relation between OTS sulcal pattern and reading ability. We expected that in line with the findings of Cachia et al.^10^ and Borst et al.^9^, participants with an interrupted left posterior OTS, in particular in its posterior portion hosting the VWFA, would have better reading performance than participants who have a continuous left OTS. We indeed did find an association between the sulcal pattern of the Left posterior OTS and reading. The association was weak and was however opposite to our expectations and of previous reports^9,10^.

Our third aim was to examine the specificity of the effect of the IPS sulcal pattern on numerical abilities as well as the specificity of the effect of the OTS sulcal pattern on reading. However, we were unable to find an effect of either the IPS sulcal pattern in numerical abilities or of the OTS sulcal pattern on reading abilities, and therefore we did not further pursue this aim.

The present study was unable to replicate a previously observed relationship between the IPS sulcal pattern and mathematical ability Roell et al.,^11^, and a previously observed association between the left posterior OTS sulcal pattern and reading^9,10^. It is unlikely that the lack of replication is due to our methods. Indeed, we followed the protocol for labeling the IPS^11^ and the OTS^9,10^ extremely closely. Our inter-rater reliability was relatively high at 80.59%. In addition, the academic measures used did not differ from the ones used in previous research. The symbolic number comparison measure and the arithmetic ability measure were very similar to the ones used in Roell et al.’s^11^ study. The Dutch one-minute test is extremely similar to the test used by both Cachia et al.^10^ and Borst et al.^9^ in their studies, as it also relied on the number of words read correctly aloud in a given time. Additionally, this measure is a very reliable reading measure and has been widely used in research.

The lack of conclusive findings, might be due to the fact that the sample in this study might have been too homogenous in terms of its academic performance. All participants came from the same grade and they were from fairly high socio-economic backgrounds. As such, it may be that the performance in the math and reading tasks may not have had enough variability to be explained by the OTS and IPS sulcal measures. Past studies have selected specific samples that were more varied in their performance. Cachia et al.’s^10^ sample consisted of participants that learnt to read as children and participants that had learnt to read as adults, thereby assuring a great variability in reading ability. In addition, the participants’ background was extremely diverse with participants from both low and high socio-economic backgrounds. In Roell et al.’s^11^ study, variability in mathematical ability was assured through a wide sample age range, as participants in the children group range from Grade 1 to Grade 4. Although by selecting heterogeneous samples the previous studies have been able to measure variability in the academic skills, the findings of these studies might have been confounded by age and SES differences in samples rather than real differences in the association between academic skills and sulcal morphology. Another possibility might be that the effects of the association between sulcal patterns and different academic skills are only visible at certain developmental stages, outside the age range tested in this study. Future studies should therefore examine specifically the possible effect of age and SES differences on the relation between sulcal morphology and academic abilities.

It may also be that there was an effect of sulcal morphology, but it was too subtle for us to pick up. Indeed, the Bayesian effects observed were all predominantly small thus pointing towards anecdotal or weak evidence. This could mean that either the effects of sulcal morphology on academic achievements are not there or are too small and much larger samples are needed to pick them up. Currently the data presented here cannot unravel this point and a study with a larger sample would be needed. Note that our sample was larger than all previously reported studies on sulcal morphology and that it was sufficiently powered to detect a medium effect size (d = 0.30, power = 0.86). However, it was not for detecting small effects (d = 0.10, power = 0.17).

Although we followed extremely closely the protocols elaborated by Borst et al.^9^, Cachia et al.^10^ and Roell et al.^11^, we were not able to replicate their results. It is important to note that although the protocols were closely followed, the distribution of the IPS and OTS sulcal patterns found in the current study were comparable to the distributions reported in other articles^9–11^. However, right IPS distribution in the current study was somewhat different from Roell et al.^11^. Likewise, the distribution of the left anterior OTS patterns was different between the current study and Cachia et al.^10^. These small differences in the distribution of sulcal patterns could explain the differences between the current study and previous ones. However, it is important to note that these differences in distribution are not particularly large.

Another possible explanation for the lack of replication is the country-based and language-based educational differences. As mentioned above, children in Flemish schools have notably high math fluency whereas children from Roell et al.’s^11^ study came from Ontario (Canada). Importantly, children from Ontario (Canada) have significantly lower than average math fluency levels^84^. Future studies should examine the potential effect of language and educational differences on the relationship between sulcal morphology and academic abilities.

A possible explanation for our findings contradicting our hypotheses and past findings^10,11^, could be the environmental background of the participants. Indeed, previous studies investigating the effect of the sulcal morphology on cognition similarly reported opposite findings in participants with different environmental backgrounds, either before birth such as twin pregnancy^85^ or after birth such as bilingualism^10,86^. Unfortunately we do not have data on bilingualism or whether the participants were twins. Future studies should control for the potential effects of bilingualism and whether the participants were twins on the relation between sulcal morphology and academic abilities.

In conclusion, our study was unable to replicate a previously observed positive association between the IPS sulcal pattern and mathematical ability Roell et al.^11^, and a previously observed positive association between the left posterior OTS sulcal pattern and reading^9,10^. It might be that such effects are only observable in heterogenous samples (as in previous work). Differences in background and the education of the current sample as compared to previous work might be another explanation. Future studies might want to explore this effect further by conducting large-scale studies with heterogeneous samples from different countries with different educational levels.

## Supplementary Information

Supplementary Information.
