# Supplementary material for: Investigating the association between variability in sulcal pattern and academic achievement
Source: Sci Rep. 2022 Jul 19;12:12323. doi: 10.1038/s41598-022-15335-y (PMC9296655; doi:10.1038/s41598-022-15335-y)
Supplement: Supplementary file 1 — Supplementary Information. [file 41598_2022_15335_MOESM1_ESM.docx]

**Supplementary Materials**

**Supplementary Section 1. Mediation Analyses**

We examined whether the symbolic number processing mediates the relationship between Left IPS sulcal morphology and arithmetic ability. Mediation analysis showed that Left IPS morphology did not significantly affect arithmetic ability, *B* = -4.85, *z*= -1.31, *p* = .18. Analysis of indirect effect revealed that symbolic ability did not significantly mediate the relationship between Left IPS and arithmetic ability, *B* = -1.33, *z*= -0.80, *p* = .41. Left IPS sulcal morphology did not affect symbolic ability, *B* = 28.74, *z*= 0.82, *p* = .40 but symbolic number ability was found to affect arithmetic ability, *B* = -0.04, *z*= -4.07, *p* < .001. Next, we analyzed whether the symbolic number processing mediated the relationship between Right IPS sulcal morphology and arithmetic ability. Mediation analysis showed that Right IPS morphology did not significantly affect arithmetic ability, *B* = -0.66, *z*= -0.18, *p* = .85. However, analysis of the indirect effect reveal that symbolic number ability significantly mediated the relationship between Right IPS and arithmetic ability, *B* = -3.83, *z*= -2.14, *p* = .03. Right IPS sulcal morphology was found to affect symbolic ability, *B* = 81.56, *z*= 2.55, *p* = .01 and symbolic number ability in turn was found to affect arithmetic ability, *B* = -0.04, *z*= -3.97, *p* < .001. The results suggest that after accounting for the mediating role of symbolic number abilities, the sulcal morphology of the Right IPS did not significantly impact arithmetic abilities, *B* = -0.66, *z*= -0.18, *p* = .85.

**Supplementary Section 2. Aim 3**

We first examined whether the sulcal pattern of the Left and Right IPS was associated with reading ability. There was no association between Left IPS and reading, *t*(87) = 1.28 , *p* = .20, and the evidence for this absence of an association was weak BF_01_ = 2.05. There was no association between Right IPS and reading, *t*(87) = 0.36 , *p* = .72, and the evidence for this absence of an association was substantial BF_01_ = 3.62.

We then examined whether the sulcal pattern of the OTS was associated with math ability. We first focused on arithmetic ability, there was no association (*t* < 1) between Left posterior and anterior OTS and arithmetic. The evidence for this absence of an association and was substantial for the Left posterior BF_01_ = 4.35 and weak for the Left anterior OTS BF_01_ = 2.51. Similarly no association between Right posterior and anterior OTS and arithmetic was found, *t* < 1 and *t*(87) = 1.02 , *p* = .31. The evidence for this absence of an association was substantial for the Left posterior, BF _01_ = 4.01 and weak for Left anterior OTS, BF_01_ = 2.79.

We then focused on symbolic number ability, we did not find an association between Left posterior and anterior OTS and symbolic number ability, *t*(84) = 1.07 , *p* = .28 and *t* < 1 respectively. The evidence for this absence of an association was weak for the Left posterior, BF _01_ = 1.36 and substantial for the Left anterior OTS BF_01_ = 3.37. Contrastingly, the Right posterior OTS was found to be significantly associated with reading ability, *t*(84) = 2.77, *p* = .006 but the evidence for this association was weak BF_01_ = 1.16. The Right anterior OTS was not found to be associated with reading ability with *t* < 1 (see *Figure 7)*, the evidence for this association was substantial BF_10_ = 4.04.

Secondly, we tested how controlling for reading ability affected the association between IPS sulcal pattern and mathematical ability. We first focused on arithmetic ability. The covariate reading ability was significantly related to arithmetic ability, *F*(1, 86) = 17.47, *p <* .001 and the evidence for this an association was strong BF_10_ = 643.15. There was no significant effect of Left IPS , *F*(1, 86) = 1.50, *p =* .22, on arithmetic ability after controlling for reading ability and the evidence for the absence of an association was weak, BF _01_ = 1.35. There was also no significant effect of Right IPS , *F*(1, 86) = 1.63, *p =* .20, on arithmetic ability after controlling for reading ability and the evidence for the absence of an association was weak, BF _01_ = 2.07. We then focused on symbolic number ability. The covariate reading ability was significantly related to arithmetic ability, *F*(1, 83) = 5.54, *p* = .02 but the evidence for this lack of an association was weak BF_10_ = 2.54. No significant effect of Left IPS, *F <* 1, on arithmetic ability after controlling for reading ability was found and the evidence for this absence of association was substantial, BF _01_ = 4.18. A mildly significant effect of Right, IPS *F*(1, 83) = 4.07, *p =* .04, on arithmetic ability after controlling for reading ability but the evidence for this an association was weak BF_10_ = 1.45.

We also tested how controlling for mathematical ability effected the association between OTS sulcal pattern and reading ability. We first controlled for arithmetic ability, again arithmetic was significantly related to reading ability, *F*(1, 86) = 21.79, *p <* .001 and the evidence for this an association was strong BF_10_ = 123.26. There was no significant effect of Left posterior OTS, *F*(1, 86) = 2.82, *p =* .09 on reading ability after controlling for arithmetic ability and the evidence for this absence of association was weak, BF _01_ = 1.62. There was no significant effect of Left anterior OTS, *F*(1, 86) = 1.22, *p =* .27 on reading ability after controlling for arithmetic ability and the evidence for this absence of association was weak, BF _01_ = 1.43. There was no significant effect of Right posterior OTS, *F*(1, 86) = 3.06, *p =* .08 on reading ability after controlling for arithmetic ability and the evidence for this absence of association was weak, BF _01_ = 1.58. No significant effect of Right anterior OTS, *F* < 1 on reading ability emerged after controlling for arithmetic ability and the evidence for this absence of association was substantial, BF _01_ = 4.03.

We then controlled for symbolic number ability, the covariate symbolic number ability was not significantly related to reading ability, *F*(1, 84) = 2.52, *p =* .11 and the evidence for this an association was substantial BF_10_ = 3.22. No significant effect of Left posterior OTS, *F*(1, 84) = 3.67, *p =* .06 on reading ability after controlling for symbolic number ability was found and the evidence for this absence of association was weak, BF _01_ = 1.61. There was no significant effect of Left anterior OTS, *F* < 1 on reading ability after controlling for symbolic number ability and the evidence for this absence of association was weak, BF _01_ = 1.81. No significant effectof Right posterior OTS, *F*(1, 84) = 3.71, *p =* .06 on reading ability was observed after controlling for symbolic number ability and the evidence for this absence of association was weak, BF _01_ = 2.00. There was no significant effect of Right anterior OTS, *F* < 1 on reading ability after controlling for symbolic number ability and the evidence for this absence of association was substantial, BF _01_ = 4.00.

Thirdly, we examined whether mathematical and reading ability are associated to the sulcal pattern of the ACC, a sulcus unrelated to both academic skills. We investigated whether the sulcal pattern of the ACC was associated with mathematical ability. We first focused on arithmetic ability. There was no association between Left and Right ACC and arithmetic, *t*(87) = 1.14 , *p* = .25, and *t* < 1 respectively. The evidence for this absence of an association weak for the Left ACC BF_01_ = 2.46 and substantial for the Right ACC BF_01_ = 3.01. We then focused on symbolic number ability. There was no association between Left and Right ACC and symbolic number ability, *t* < 1, and *t* < 1 respectively. The evidence for this absence of an association for both the Left and Right ACC was substantial BF_01_ = 3.89 and BF_01_ = 4.03 respectively.

We then investigated whether the sulcal pattern of the ACC was associated with reading ability. There was no association between Left and Right ACC and reading, *t*(87) = 1.29 , *p* = .19, and *t* < 1 respectively. The evidence for this absence of an association for both the Left and Right ACC was weak BF_01_ = 2.00 and BF_01_ = 3.58 respectively.

Finally, we also tested whether a relationship between the sulcal pattern of the IPS and that of the OTS could be found. The sulcal pattern of the IPS was not found to be correlated with the sulcal pattern of the OTS, see Supplementary Table 1.

*Supplementary Table 1.* Pearson correlations among IPS and OTS sulcal patterns

|  | 1 | 2 | 3 | 4 | 5 |
| --- | --- | --- | --- | --- | --- |
| 1. Left IPS | - | - | - | - | - |
| 1. Right IPS | -.01 | - | - | - | - |
| 1. Left posterior OTS | -.12 | -.06 | - | - | - |
| 1. Left anterior OTS | .00 | .05 | -.25 | - | - |
| 1. Right posterior OTS | -.06 | .12 | .04 | .00 | - |
| 1. Right anterior OTS | .04 | -.05 | .00 | -.02 | -.19 |

**Supplementary Section 3. Deviations from the Preregistered Study**

Below are all the all the deviations from the preregistered study and the reasoning behind them:

1. We used only 2 datasets rather than 3, as the T1 MRI of the third dataset had too much movement for the Brainvisa to extract the relevant information and create three-dimensional mesh-based reconstructions of the cortical folds.
2. We modified our statistical approach from a step-wise approach to a model in which database, IQ and sex were a priori considered as cofounding variables. In the preregistered step-wise statistical model, the relationship between the sulcal morphology and the cognitive scores are first explored using simple t-tests. The effect of a priori covariates (IQ, database and sex) on cognitive scores are also explored. Then, if an association is found between the cognitive score and the sulcal morphology, subsequent analyses test whether the association holds when a priori covariates (IQ, sex and database) are included. This approach was modified upon the recommendations from a Reviewer as this approach is prone to provide false negative findings as each covariate may not have a significant effect on the cognitive scores, but may explain an important part of the variance when considered altogether and with the sulcal variable. In addition, the proposed two-step approach does not allow investigating the possible interactions between the cofounding covariates and the sulcal variables.
